# Anomalous Aortic Origin of the Right Coronary Artery: Invasive Haemodynamic Assessment in Adult Patients With High-Risk Anatomic Features

**DOI:** 10.1016/j.cjcpc.2023.03.001

**Published:** 2023-03-08

**Authors:** Diederick B.H. Verheijen, Anastasia D. Egorova, Monique R.M. Jongbloed, Frank van der Kley, Dave R. Koolbergen, Mark G. Hazekamp, Hildo J. Lamb, J. Wouter Jukema, Philippine Kiès, Hubert W. Vliegen

**Affiliations:** a Department of Cardiology, CAHAL, Center for Congenital Heart Disease Amsterdam-Leiden, Leiden University Medical Center, Leiden, the Netherlands; b Department of Cardiology, Leiden University Medical Center, Leiden, the Netherlands; c Department of Anatomy and Embryology, Leiden University Medical Center, Leiden, the Netherlands; d Department of Cardiothoracic Surgery, CAHAL, Center for Congenital Heart Disease Amsterdam-Leiden, Leiden University Medical Center, Leiden, the Netherlands; e Department of Cardiothoracic Surgery, Leiden University Medical Center, Leiden, the Netherlands; f Department of Radiology, Leiden University Medical Center, Leiden, the Netherlands; g Netherlands Heart Institute, Utrecht, the Netherlands

## Abstract

**Background:**

Anomalous aortic origin of a right coronary artery (AAORCA) with an interarterial course merits further evaluation; however, robust risk assessment strategies for myocardial ischemia and sudden cardiac death are currently lacking. The aim of this study is to explore the potential role of fractional flow reserve (FFR), instantaneous wave-free ratio (iFR), and intravascular ultrasound (IVUS) in patients with AAORCA.

**Methods:**

Consecutive adult patients with AAORCA with an interarterial course were included. Computed tomography angiography, noninvasive ischemia detection, and FFR, iFR, and IVUS were performed at baseline and during adrenaline-induced stress. External compression was evaluated with IVUS.

**Results:**

Eight patients (63% female, mean age: 53 ± 9.5 years) were included. Five patients (63%) were symptomatic, and computed tomography angiography revealed high-risk anatomy of the AAORCA in all patients. Only in 1 (12.5%) patient FFR and iFR were positive; however, this was attributed at large to concomitant diffuse atherosclerosis. In 2 of 8 (25%), IVUS revealed external compression; however, the ostial coronary surface area remained unchanged. In all patients, a conservative treatment strategy was pursued. During a mean follow-up of 29.3 months (standard deviation ±2.6 months), symptoms spontaneously disappeared in 4 of 5 (80%) and no adverse cardiac events occurred in any of the patients.

**Conclusions:**

Despite the presence of high-risk anatomy in all patients, none had proven ischemia prompting a conservative treatment strategy. No adverse cardiac events occurred during follow-up, and in the majority of patients, symptoms spontaneously disappeared. Therefore, FFR, iFR, and IVUS with pharmacologic stress merit further investigation and might contribute to ischemia-based risk stratification and management strategies in adult patients with AAORCA.

## Résumé

**Contexte:**

L’anomalie de naissance de l’artère coronaire droite à partir de l’aorte (AAORCA, anomalous aortic origin of a right coronary artery) combinée à un trajet interartériel mérite un examen plus approfondi. Cependant, on observe à l’heure actuelle des lacunes en ce qui a trait à l'emploi de stratégies fiables d’évaluation du risque d’ischémie myocardique et de mort subite d’origine cardiaque. L’objectif de cette étude est d’examiner le rôle potentiel de la mesure de la réserve coronarienne (MRC), de l’évaluation du rapport instantané sans onde (iFR, *instantaneous wave-free ratio*) et de l’échographie intravasculaire chez des patients présentant une AAORCA.

**Méthodologie:**

Des cas de patients adultes consécutifs présentant une AAORCA combinée à un trajet interartériel ont été inclus à l’étude. Une angiographie par tomodensitométrie (TDM), une détection non invasive de la présence d’une ischémie, la MRC, l’évaluation de l’iFR et l’échographie intravasculaire ont été effectuées au début de l’étude ainsi que lors d’un stress induit par l’adrénaline. La compression externe a également été évaluée au moyen d’une échographie intravasculaire.

**Résultats:**

Huit patients (63 % de sexe féminin; âge moyen de 53 ans ± 9,5 ans) ont participé à l’étude. Cinq patients (63 %) présentaient des symptômes, et l’angiographie par TDM a révélé une AAORCA à risque élevé chez tous les patients. Les résultats de la MRC et de l’évaluation de l’iFR étaient positifs chez seulement un patient (12,5 %), ce qui est attribuable en majeure partie à une athérosclérose diffuse concomitante. Chez deux patients (25 %), l’échographie intravasculaire a montré une compression externe de l’artère coronaire droite même si l’aire de l’ostium de l’artère n’avait pas changé. Une stratégie thérapeutique prudente a été employée pour tous les patients. Pendant la période de suivi qui a duré en moyenne 29,3 mois (écart-type : ± 2,6 mois), les symptômes se sont résorbés de manière spontanée chez quatre des cinq patients (80 %), et aucun événement cardiaque indésirable n’est survenu.

**Conclusion:**

Malgré une anatomie à risque élevé chez tous les patients, aucun d’entre eux ne présentait une ischémie connue, ce qui justifiait une stratégie thérapeutique prudente. Aucun événement cardiaque indésirable n’est survenu durant la période de suivi, et les symptômes se sont résorbés de manière spontanée chez la majorité des patients. À la lumière de ces renseignements, la MRC, l’évaluation de l’iFR et l’échographie intravasculaire lors d’un stress pharmacologique devraient faire l’objet d’autres études et pourraient éventuellement être utiles dans la stratification du risque d’ischémie et dans le choix des stratégies de prise en charge des patients adultes présentant une AAORCA.

Anomalous aortic origin of a coronary artery (AAOCA), also often referred to as anomalous origination of a coronary artery from the opposite sinus of Valsalva (ACAOS), is a congenital heart defect in which a coronary artery arises from an aberrant position in the aorta. The prevalence of AAOCA is 0.1%-1% in the general population.^1^, ^2^, ^3^ AAOCA with a retroaortic or prepulmonic course usually does not provoke myocardial ischemia and is therefore generally considered benign.^4^^,^^5^ In contrast, AAOCA with an interarterial or septal course is considered potentially malignant as these variants are associated with an increased risk of myocardial ischemia and sudden cardiac death (SCD).^6^^,^^7^ AAOCA is reported to be accountable for 7%-17% of SCD in young athletes.^8^ Based on postmortem studies, this is particularly the case in patients who are younger than 35 years.^9^^,^^10^ Obstructive coronary artery disease is the leading cause of SCD in older patients, although AAOCA still does carry an SCD risk in selected individuals above the age of 35.^11^^,^^12^

Identifying individuals with AAOCA at risk for SCD remains challenging, and recent European Society of Cardiology (ESC) guidelines and American Heart Association (AHA) guidelines stratify a number of anatomic “high-risk” features, including high orifice take-off, ostial stenosis, slitlike/fish-mouth–shaped orifice shape, acute-angle take-off, a long intramural course or interarterial course, and hypoplasia of the proximal coronary artery.^13^^,^^14^ Computed tomography angiography (CTA) is the imaging technique of choice for adults with AAOCA.^13^^,^^14^ One of the main limitations is, however, the inability of CTA to capture the dynamic changes in ostial anatomy and haemodynamics during or shortly after peak exercise, which is when most SCD in patients with AAOCA have been documented.^15^

The ESC guidelines^13^ provide clear treatment recommendations for patients with AAOCA and typical angina symptoms and evidence of stress-induced myocardial ischemia in the matching territory or with high-risk anatomy. However, treatment advice for patients who present with anomalous aortic origin of a right coronary artery (AAORCA) with an interarterial course and high-risk anatomic features but without evidence for ischemia (a common presentation of patients with AAOCA) is not indisputable.^13^ In our tertiary referral centre for patients with AAOCA, the treatment strategy for the last-mentioned group of patients, of whom only the minority present with typical angina complaints,^16^ historically often resulted in surgical intervention. This was substantiated by the presence of high-risk anatomic features and lack of validated noninvasive ischemia detection methods. An improvement of the diagnostic workup and ischemia-based treatment strategies in patients with AAOCA might contribute to a more individualized approach.

For the assessment of myocardial ischemia during exercise, nonpharmacologic functional “stress” imaging is recommended.^13^ Invasive assessment using fractional flow reserve (FFR), instantaneous wave-free ratio (iFR), and intravascular ultrasound (IVUS) has proven their incremental value in guiding the management of patients with obstructive coronary artery disease.^17^, ^18^, ^19^ These techniques are technically feasible in the patients with AAOCA, and it is therefore of interest whether they can contribute to the individual risk stratification in this group.^20^, ^21^, ^22^, ^23^, ^24^

In the current study, we describe the first experience in our tertiary referral centre with invasive functional testing incorporating FFR, iFR, and IVUS performed at baseline and during pharmacologic stress in conjunction with the regular diagnostic workup in adult patients with an anomalous right coronary artery with anatomic high-risk features.

## Methods

In this retrospective cohort study, all consecutive adult (≥18 years old) patients with an AAORCA with an interarterial course who were referred to our tertiary referral centre between September 2019 and March 2020 and who underwent invasive functional and haemodynamic testing using FFR, iFR, and IVUS alongside with the regular noninvasive workup were included. Demographic and clinical data were retrieved from the electronic health record systems (EPD-Vision; Leiden University Medical Center, Leiden, the Netherlands and HiX; Chipsoft, Amsterdam, the Netherlands).

Complaints at presentation were categorized according to the 2021 AHA/American College of Cardiology (ACC) guideline for the evaluation and diagnosis of chest pain by 3 dedicated adult congenital heart disease (ACHD) cardiologists.^25^ Chest pain was deemed as either cardiac, possibly cardiac, or noncardiac. Anatomic features as defined in the ESC guidelines were examined on high-resolution CTA scans.^13^ Ischemia detection consisted of a bicycle exercise test and further noninvasive detection of ischemia at discretion of the referring cardiologist. In addition to the regular diagnostic workup, the patient underwent coronary angiography and invasive evaluation including FFR, iFR, and IVUS.^26^, ^27^, ^28^ FFR and IVUS were carried out according to the previously published protocol by Driesen et al.,^23^ who showed that these measurements seem feasible and appear to be safe as no events were observed during a median follow-up of 37 months. The management strategy was decided on after the discussion in the ACHD heart team consisting of ACHD-cardiologists, interventional cardiologists, and cardiothoracic surgeons specialized in ACHD and AAOCA.

### Computed tomography angiography

The CTA scans had a slice thickness of 0.5-0.6 mm, were ECG triggered, and were performed at a target heart rate of 65 beats per minute (bpm) or less. To achieve this, patients received up to 100 mg of metoprolol orally before the scan. Sublingual nitroglycerin (single dose of 0.4 mg) was administered before the scan according to the Society of Cardiovascular Computed Tomography guidelines for the performance and acquisition of coronary CTA.^29^ The images were analysed in the diastolic phase at 60%-75% of the R-R interval, at the width of 1200 Hounsfield units (HU) and the level of 300 HU.

**Figure 1:**
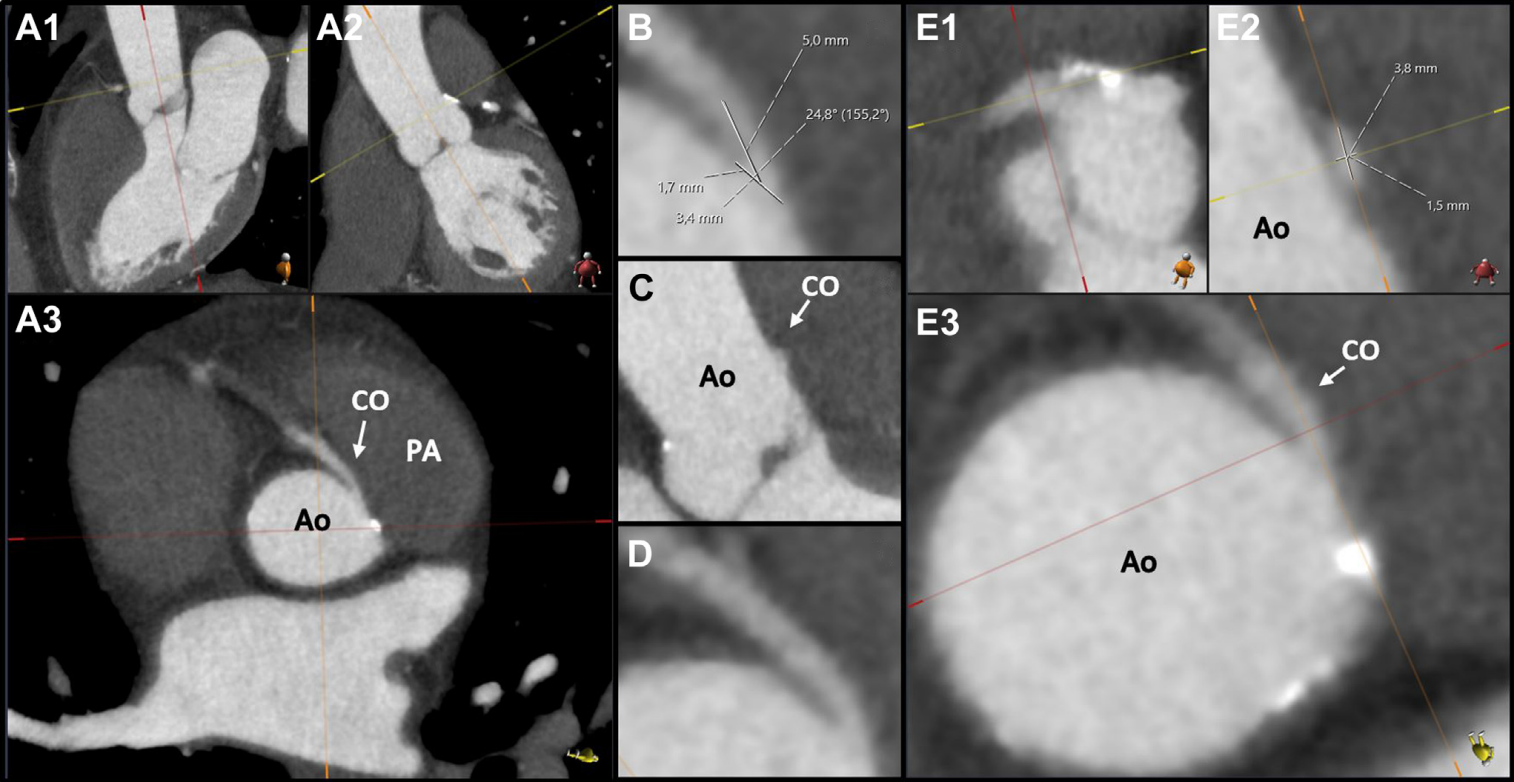
CTA analysis approach. (**A**_**1**_**-A**_**3**_) Three-dimensional multiplanar reconstruction with double oblique planes perpendicular to the aortic annulus. (**B**) Acute-angle take-off measurement. (**C**) Orifice take-off in relation to the sinotubular junction. (**D**) Assessment of an intramural course and its length. (**E**_**1**_**-E**_**3**_) Orifice shape measurement. Ao, aorta; CTA, computed tomography angiography; CO, coronary ostium of the AAORCA; PA, pulmonary artery.

The coronary anatomy was classified according to the Leiden Convention coronary coding system.^30^ Cardiac dominance was determined by the coronary artery from which the posterior descending artery originated and was characterized as either left, right, or codominant.^31^ High-risk anatomic features associated with myocardial ischemia, as stated by the ESC and AHA/ACC guidelines, were analysed according to standard practice (Fig. 1).^13^ First, a 3-dimensional multiplanar reconstruction with double oblique planes was created parallel to the centreline of the aorta and perpendicular to the leaflets of the aortic valve (Fig. 1A). In this view, the course (Fig. 1A), the acute-angle take-off (Fig. 1B), the orifice take-off in relation to the sinotubular junction (Fig. 1C), and the suspicion for an intramural course (Fig. 1D) were determined. For the analysis of the orifice shape and the presence of hypoplasia of the proximal coronary artery, a 3-dimensional multiplanar reconstruction with double oblique planes parallel to the centreline of the anomalous coronary artery and perpendicular to the coronary ostium was generated (Fig. 1E). In this view, the orifice shape was quantified. To assess hypoplasia of the proximal coronary artery, the ostium shape was measured distally and compared with the proximal measurements. The course of the anomalous coronary artery was considered interarterial if the anomalous coronary artery passed between the ascending aorta and the pulmonary artery (above the level of the pulmonary valve). A take-off angle of <45° was regarded an acute angle take-off.^32^ The orifice take-off in relation to the sinotubular junction was categorized according to the classification as previously described.^2^ An orifice take-off >1 cm above the sinotubular junction was defined as a high take-off. The orifice was considered slitlike if the orifice width was ≤50% of the orifice height, and proximal narrowing was defined as the proximal width being ≤50% of the distal width.^33^ An intramural course was suspected if the interluminal space was <0.95 mm at 2 mm from the ostium.^34^

### Coronary angiography with invasive assessment using FFR, iFR, and IVUS

All the patients underwent coronary angiography to evaluate concomitant obstructive coronary artery disease. Stenosis of >50% in the left main coronary artery or >70% in a major coronary artery was considered to be significant.^35^ FFR and iFR measurements were obtained using a pressure guide wire (Prime Wire; Volcano Imaging System Philips Healthcare, San Diego, CA). FFR and iFR were performed according to the common procedure for atherosclerotic lesions using femoral access.^36^ FFR and iFR were regarded positive at the cutoff values of ≤0.80 for FFR and ≤0.89 for iFR.^37^ To evaluate the potential dynamic effects of the anomalous coronary artery, FFR during adenosine infusion (140 mcg/min/kg intravenous [iv]) and iFR after an intracoronary bolus of nitroglycerin (0.2 mg intracardiac [ic]), as well as FFR and iFR during adrenaline infusion (adrenaline 0.025-0.1 mg iv), were performed.^38^ The adrenaline infusion dose was individualized to attain a target heart rate of >130 bpm or a systolic blood pressure of >150 mm Hg.^23^

**Figure 2:**
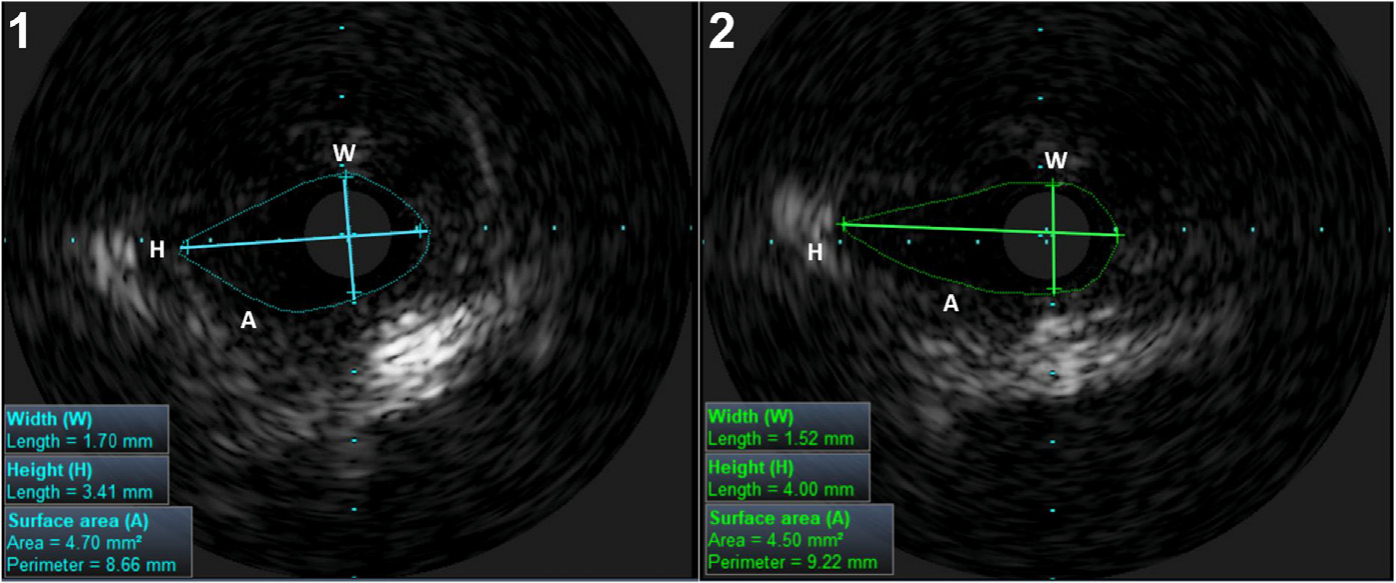
Intravascular ultrasound (IVUS) analysis approach. IVUS measurements of the height (H), width (W), and coronary surface area (A) at baseline (**1**) and during adrenaline-induced stress (**2**) for the assessment of the “width/height ratio” to determine the coronary vessel shape at baseline, the “width/height ratio during adrenaline-induced stress”/“width/height ratio at baseline,” to evaluate external compression and to estimate the change in the coronary surface area.

IVUS was performed to evaluate the dimensions of the proximal anomalous coronary artery at baseline after the intracoronary administration of nitroglycerin (0.2 mg ic) and subsequently with adrenaline infusion (adrenaline 0.025-0.1 mg iv). A dedicated IVUS imaging system (S5 Imaging System; Volcano Corporation, Rancho Cordova, CA) was used. The definitions of a slitlike orifice on IVUS and on CTA are identical and were thus defined by the width/height ratio (Fig. 2). To assess potential external compression, the “width/height ratio during pharmacologic stress” was divided by the “width/height ratio at baseline.” A lower absolute value of the ratio denotes a more severe degree of external compression during pharmacologic stress.

### Statistical analysis

Statistical analysis was performed using SPSS (version 25; SPSS Inc, Chicago, IL). Categorical data were reported as numbers and percentages. The Shapiro-Wilk test was performed to evaluate the normal distribution of continuous data. Normally distributed continuous data were presented as mean ± standard deviation (SD). Differences in the coronary surface area and width/height ratio at baseline and during adrenaline infusion on IVUS were assessed with a paired-samples *t*-test. A *P* value of <0.05 was considered to be statistically significant.

## Results

Eight patients with an anomalous aortic origin of a right coronary artery (AAORCA) with an interarterial course were identified (Table 1). Five (63%) patients were female, and the mean age was 53 years (SD ±9.5 years). One patient had a history of myocardial infarction in the effluent territory of the AAORCA, which was attributed to obstructive coronary artery disease of the mid RCA segment and treated with a primary percutaneous coronary intervention. Three (38%) patients presented with possible cardiac chest pain and 2 (25%) patients with noncardiac chest pain. In 3 (38%) patients, the diagnosis of the anomalous coronary artery was an incidental finding on a CTA performed for another indication. Transthoracic echocardiography showed a normal left and right ventricular function in 7 (88%) patients. In 1 patient, it showed a moderately reduced left ventricular function secondary to a prior myocardial infarction in a nonanomalous coronary artery territory. The mean follow-up after evaluation was 29.3 months (SD ±2.6 months).

**Table 1:** Patient demographics and diagnostic results

Study number	Age	Sex	Relevant medical history	Relevant medication at baseline	Symptoms at diagnosis	Echo	Bicycle exercise test	Ischemia in the matching territory: noninvasive detection	Final treatment advice
1	45	Male	None	None	Possible cardiac chest pain	Normal	NP	Nuclear SPECT (physical stress): negative	Conservative
2	64	Female	HT, vascular claudication	ACEI, statin	Noncardiac pain	Normal	NP	Nuclear SPECT (adenosine): negative	Conservative
3	57	Female	HT, DM2, ocular sarcoidosis	ARB	Incidental finding	Normal	Negative	Nuclear SPECT (adenosine): negative	Conservative
4	49	Female	Asthma	None	Possible cardiac chest pain	Normal	Negative	Echo (dobutamine): negative	Conservative
5	50	Female	None	None	Possible cardiac chest pain	Normal	Negative	Nuclear SPECT (physical stress): negative	Conservative
6	52	Female	None	None	Noncardiac pain	Normal	Negative	Nuclear SPECT (physical stress): negative	Conservative
7	69	Male	OHCA due to IPL infarction, PCI RCx-MO	BB, ACEI, statin	Incidental finding	Moderate LV function	NP	Nuclear SPECT (adenosine): negative	Conservative
8	41	Male	AVNRT ablation, NSTEMI with PCI RCA and RCx	BB, ACEI, statin	Incidental finding	Normal	Inconclusive	MRI (dobutamine): negative	Conservative

ACEI, angiotensin-converting enzyme inhibitors; ARB, angiotensin receptor blockers; AVNRT, atrioventricular-nodal re-entry tachycardia; BB, β-blocker; DM2, diabetes mellitus type 2; HT, hypertension; IPL, inferoposterolateral; LV, left ventricular; MO, obtuse marginal artery; MRI, magnetic resonance imaging; NP, not performed; NSTEMI, non-ST-elevation myocardial infarction; OHCA, out of hospital cardiac arrest; PCI, percutaneous coronary intervention; RCA, right coronary artery; RCx, ramus circumflex; SPECT, single photon emission computed tomography; statin, HMG-CoA reductase inhibitors.

### Computed tomography angiography

All 8 patients had an AAORCA with an interarterial course, classified as “2R∗,LCx” according to the Leiden Convention coronary coding system, and a right-dominant circulation (Table 2). Of these, 6 (75%) had a slitlike orifice, 6 (75%) had an acute angle take-off, and 6 (75%) had hypoplasia of the proximal coronary artery. The mean coronary take-off angle was 32.4° (SD ±13.8°). None of the patients had an orifice take-off of >1.0 cm above the sinotubular junction, and 7 of 8 (88%) had a high degree of suspicion of an intramural course based on the CTA. Overall, 2 of 8 (25%) patients had 3 high-risk anatomic features, 3 of 8 (37.5%) had 4 high-risk anatomic features, and 3 of 8 (37.5%) had 5 high-risk anatomic features as defined by the current ESC guidelines (Table 2).

**Table 2:** Anatomic features on CTA

Patient	Coronary anatomy according to the Leiden Convention	Orifice shape	Coronary take-off angle (°)	Hypoplasia of the proximal coronary artery	High take-off to STJ	Take-off in relation to the PuV	Suspicion of an intramural course (ILS < 0.95 mm at 2 mm from the ostium)	Coronary artery disease
1	2R∗,LCx	Slitlike	25	Yes	No	Same	Yes	No significant lesions
2	2R∗,LCx	Slitlike	25	Yes	No	Above	Yes	No significant lesions
3	2R∗,LCx	Oval	30	No	No	Same	Yes	No significant lesions
4	2R∗,LCx	Slitlike	29	No	No	Same	No	No significant lesions
5	2R∗,LCx	Slitlike	60	Yes	No	Under	Yes	No significant lesions
6	2R∗,LCx	Slitlike	27	Yes	No	Same	Yes	No significant lesions
7	2R∗,LCx	Oval	17	Yes	No	Above	Yes	No significant lesions
8	2R∗,LCx	Slitlike	46	Yes	No	Under	Yes	No significant lesions

CTA, computed tomography angiography; ILS, interluminal space; PuV, pulmonary valve; STJ, sinotubular junction.

### Noninvasive detection of ischemia

In 5 (63%) patients, bicycle ergometry was performed in the diagnostic workup. Of these 5 patients, 4 (80%) had a negative bicycle exercise test (no signs of ischemia), and in 1 patient (20%), the test was deemed inconclusive due to failure to reach the target heart rate. A nuclear exercise stress test was performed in 3 (37.5%) patients, a nuclear adenosine stress test in 3 (37.5%) patients, dobutamine stress magnetic resonance imaging in 1 (12.5%) patient, and dobutamine stress echocardiography in 1 (12.5%) patient. All patients had a negative noninvasive ischemia detection test.

### Coronary angiography and invasive pressure measurements

Coronary angiography showed no significant atherosclerotic lesions in the anomalous coronary artery in all patients (Table 3). iFR at baseline was measured in 7 of 8 (87.5%) patients, FFR with adenosine infusion was measured in all patients, FFR with adrenaline infusion in all patients, and iFR with adrenaline infusion in 5 of 8 (62.5%) patients. Baseline iFR and FFR measurements were negative for ischemia in 6 of 7 (86%) patients and 7 of 8 (88%) patients, respectively. During pharmacologic stress, FFR was negative in all patients and iFR remained negative in 4 of 5 (80%) patients. All measurements positive for ischemia were observed in 1 patient, patient 7, who showed a positive iFR at baseline, FFR during adenosine infusion, and iFR after adrenaline infusion with values of 0.88, 0,67, and 0.82, respectively. FFR during adrenaline was negative with a value of 0.81. However, in this case, the positive measurements were at large attributed to the concomitant diffuse atherosclerosis in the mid segment of the AAORCA, in addition to a possible AAORCA component.

**Table 3:** Coronary angiography and invasive detection of ischemia with FFR and iFR

Patient	At baseline	Adenosine infusion	Adrenaline infusion		Coronary artery disease
	iFR	FFR	FFR	iFR	
1	NP	0.85	0.99	NP	No atherosclerosis
2	0.98	0.88	0.97	NP	<30% stenosis mid RCA
3	0.98	0.92	0.95	0.94	No atherosclerosis
4	1.0	0.86	0.99	0.98	No atherosclerosis
5	0.97	0.94	0.95	0.95	No atherosclerosis
6	0.99	0.82	0.95	0.96	No atherosclerosis
7	0.88∗	0.67∗	0.81	0.82∗	Diffuse atherosclerosis mid RCA
8	0.94	0.83	0.87	NP	No atherosclerosis

FFR, fractional flow reserve; iFR, instantaneous wave-free ratio; NP, not performed; RCA, right coronary artery.

∗ Positive outcome for ischemia.

### Intravascular ultrasound

**Figure 3:**
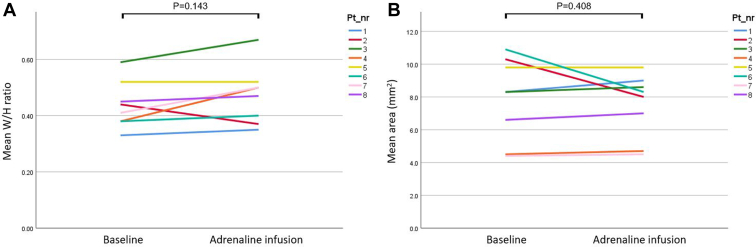
(**A**) Intravascular ultrasound (IVUS) measurements of the mean width/height (W/H) ratio at baseline and during adrenaline-induced stress. (**B**) IVUS measurements of the mean coronary surface area (mm^2^) at baseline and during adrenaline-induced stress.

IVUS at baseline and during adrenaline-induced pharmacologic stress was performed in all patients (Supplemental Table S1). IVUS at baseline showed a slitlike orifice in 6 of 8 (75%) patients. The mean width/height ratio at baseline and during adrenaline infusion was 0.44 (SD ±0.08) and 0.47 (SD ±0.10) (*P* = 0.143), respectively. The mean coronary surface area at baseline and during adrenaline infusion was 7.5 mm^2^ (SD ±2.0 mm^2^) and 7.9 mm^2^ (SD ±2.5 mm^2^) (*P* = 0.408), respectively. In 2 of 8 (25%) patients, the “width/height (W/H)-ratio assessed during adrenaline infusion” divided by the “W/H-ratio at baseline” was 0.8, indicating external compression; however, the coronary surface area remained similar in these patients (4.7 vs 4.5 mm^2^ and 4.5 vs 4.4 mm^2^; Figure 3).

### Treatment decision and follow-up

In all patients, a conservative treatment strategy was advised by the dedicated multidisciplinary ACHD team. None of the patients underwent pharmacologic treatment modifications. One patient with possible cardiac complaints due to poorly regulated hypertension was initiated on a calcium channel blocker (amlodipine). In all 3 patients who presented with possible cardiac complaints, the symptoms spontaneously disappeared after the workup and reassurance of the treating cardiologist. In the one patient who presented with noncardiac chest pain, the symptoms also disappeared, and in the other patient, the symptoms spontaneously diminished. No major cardiac adverse events were observed during follow-up in any of the patients.

## Discussion

In this retrospective observational study, we found that, despite the high-risk anatomic features present in all of our patients with AAORCA, none of the patients had proven ischemia with the additional functional measurements prompting a conservative treatment strategy. During 2.5 years of follow-up in 80% of the patients, the symptoms spontaneously disappeared. Moreover, during the 2.5 years of follow-up (on top of the mean age of 53 years at baseline), no adverse cardiac events occurred. We believe that this knowledge could support a more conservative treatment strategy for a selected group of (paediatric) patients and that FFR, iFR, and IVUS may contribute to the diagnostic workup in adult and potentially paediatric patients with AAORCA by providing additional functional information on ischemia in the presence of multiple anatomic high-risk features.

All patients met the criteria of having “no typical angina,” “AAORCA with an interarterial course,” “high-risk anatomy,” and “negative noninvasive ischemia detection,” which is a common presentation for adult patients with AAORCA. For this specific group of patients, the ESC guidelines do not provide clear management advice.^13^ The AHA/ACC guidelines advise the consideration of both surgical intervention and continued observation with class of recommendation IIb and level of evidence B-nonrandomized.^14^

In asymptomatic patients, surgical treatment addresses the potential risk for acute myocardial infarction and SCD. However, numerous studies have demonstrated that AAOCA-related SCD predominantly occurs in patients <35 years old and in the setting of an anomalous aortic origin of a left coronary artery.^9^^,^^10^ The potential individual benefit of intervention in adults >35 years old with AAORCA anatomy is yet poorly substantiated. Several aspects are to be considered in light of this. First of all, if AAORCA variant is highly malignant, presumably this would result in complaints or complications during exercise earlier in life. This should particularly be considered in the context of both progressive decline in physical activity with age and decreasing peak heart rate at maximal exercise capacity at an older age, resulting in a decreased myocardial oxygen demand.^39^, ^40^, ^41^, ^42^, ^43^, ^44^, ^45^ Furthermore, arterial compliance decreases with aging,^46^ potentially limiting the risk of external compression of the AAORCA in between the aorta and pulmonary artery. A short-term follow-up study that included patients with AAORCA in whom a conservative treatment regimen was followed reported no complications related to the AAORCA.^47^

However, several case reports of adult patients of >35 years of age with AAROCA presenting with SCD have been described.^48^, ^49^, ^50^ Besides, to our knowledge, long-term follow-up in patients with AAORCA with conservative treatment has not been reported to date. Subsequently, surgical treatment, especially “the unroofing procedure,” is generally regarded effective, with relatively low complication rates.^51^, ^52^, ^53^ Therefore, an individual risk-benefit analysis remains important.

All patients in the current report had at least 3 high-risk anatomic features, but lacked to show any resulting myocardial ischemia on elaborate testing.

Multiple opportunities for improvement in the diagnostic workup for patients with AAOCA can be insinuated. The current ESC guidelines recommend nonpharmacologic functional imaging; however, in practice, many centres still implement adenosine, dipyridamole, or regadenoson obstructive coronary artery ischemia detection protocols for the analysis of patients with AAOCA.^13^ It should be noted that given the principally different physiological mode of action (ie, coronary microcirculation vasodilation and hyperaemia), these noninvasive protocols address mechanisms different from those expected to play a critical role in exercise-induced AAOCA-related ischemia.^26^ To illustrate, in the current cohort, in 25% of the patients, noninvasive ischemia detection was performed with adenosine-induced stress. If physical exercise testing is not available or feasible in individual patients, dobutamine and adrenaline are the pharmacologic agents of choice to best simulate physical stress and evaluate the dynamic component of AAOCA.^26^

In addition, FFR and IVUS can be performed to evaluate the fixed and dynamic components of AAOCA.^23^^,^^54^ Driesen et al.^23^ first described their experience with FFR and IVUS during dobutamine or adrenaline infusion in 30 patients with AAOCA. The study illustrated the feasibility and safety of this approach and described that orifice shape and (dynamic) coronary compression could be evaluated with IVUS and FFR. IVUS and FFR data significantly contributed to the clinical decision-making, and no serious adverse events occurred during a median follow-up of 37 months.

Moreover, to our knowledge, first presented in this study, iFR can improve the diagnostic workup in adult patients with AAORCA and is potentially even superior to FFR. Namely, iFR addresses the pressure gradient during a part of the diastole when the microvascular resistance is low.^36^ Therefore, no additional medication is needed to perform iFR in a resting baseline state or during pharmacologic stress with adrenaline or dobutamine. In contrast, FFR measures the pressure gradient during the full cardiac cycle and should therefore be performed during adenosine infusion.^36^ However, adenosine cannot be combined with pharmacologic stress with adrenaline or dobutamine. Hence, iFR measurements during pharmacologic stress with adrenaline or dobutamine might be more reliable than FFR. In this cohort, iFR at baseline and iFR with adrenaline testing were added to the invasive evaluation. iFR at baseline and FFR adenosine were performed to evaluate the fixed component and FFR (without adenosine) and iFR, both during adrenaline, to evaluate the dynamic component.^21^^,^^26^

### Study limitations

The findings of this study should be interpreted in light of the small cohort of patients. This is reflective of the rarity of the condition. However, to our knowledge, this is the first study presenting consecutive patients with AAORCA and an interarterial course, in whom a diagnostic workup including iFR measurements was performed. Secondly, as data were retrieved retrospectively, iFR measurements were not structurally performed (baseline and stress) in all patients as iFR measurements were introduced at this time in our centre. Furthermore, it is not clear whether invasive measurements only further substantiated conservative management or have in fact guided the treatment decision. Moreover, FFR and iFR cutoff values are based on studies performed in patients with obstructive coronary artery disease. So far, these cutoff values have not been validated in patients with AAOCA. To address these 3 issues, “The first multicentre study on coronary anomalies in the Netherlands: MuSCAT” has been initiated by our tertiary centre in which evidence-substantiated recommendations for diagnostic workup, treatment, and follow-up of patients with AAOCA are currently investigated.^38^

## Conclusions

Despite the presence of multiple high-risk anatomic features in all patients with AAORCA, none of the patients had proven ischemia with the additional functional measurements prompting a conservative treatment strategy. There were no adverse cardiac events during follow-up, and 80% of patients showed spontaneous disappearance of symptoms. Therefore, the potential of invasive evaluation integrating FFR, iFR, and IVUS with pharmacologic stress merits further investigation and might contribute to ischemia-based risk stratification and management strategies in adult patients with AAOCA. We believe that this knowledge can also be of potential interest for paediatric cardiologists, paediatric radiologists, and paediatric cardiothoracic surgeons as this could support a more conservative treatment strategy for a selected group of (paediatric) patients.

## References

[1] Brothers J.A., Frommelt M.A., Jaquiss R.D.B. (2017). Expert consensus guidelines: anomalous aortic origin of a coronary artery. J Thorac Cardiovasc Surg.

[2] Mery C.M., Lawrence S.M., Krishnamurthy R. (2014). Anomalous aortic origin of a coronary artery: toward a standardized approach. Semin Thorac Cardiovasc Surg.

[3] Angelini P., Cheong B.Y., Lenge De Rosen V.V. (2018). High-risk cardiovascular conditions in sports-related sudden death: prevalence in 5,169 schoolchildren screened via cardiac magnetic resonance. Tex Heart Inst J.

[4] Grani C., Benz D.C., Schmied C. (2016). Prevalence and characteristics of coronary artery anomalies detected by coronary computed tomography angiography in 5 634 consecutive patients in a single centre in Switzerland. Swiss Med Wkly.

[5] Roberts W.C., Kragel A.H. (1988). Anomalous origin of either the right or left main coronary artery from the aorta without coursing of the anomalistically arising artery between aorta and pulmonary trunk. Am J Cardiol.

[6] Frescura C., Basso C., Thiene G. (1998). Anomalous origin of coronary arteries and risk of sudden death: a study based on an autopsy population of congenital heart disease. Hum Pathol.

[7] Cheitlin M.D., De Castro C.M., McAllister H.A. (1974). Sudden death as a complication of anomalous left coronary origin from the anterior sinus of Valsalva, a not-so-minor congenital anomaly. Circulation.

[8] Schiavone M., Gobbi C., Gasperetti A., Zuffi A., Forleo G.B. (2021). Congenital coronary artery anomalies and sudden cardiac death. Pediatr Cardiol.

[9] Bohm P., Scharhag J., Meyer T. (2016). Data from a nationwide registry on sports-related sudden cardiac deaths in Germany. Eur J Prev Cardiol.

[10] Basso C., Maron B.J., Corrado D., Thiene G. (2000). Clinical profile of congenital coronary artery anomalies with origin from the wrong aortic sinus leading to sudden death in young competitive athletes. J Am Coll Cardiol.

[11] Finocchiaro G., Papadakis M., Robertus J.L. (2016). Etiology of sudden death in sports: insights from a United Kingdom Regional Registry. J Am Coll Cardiol.

[12] Wu Q., Zhang L., Zheng J. (2016). Forensic pathological study of 1656 cases of sudden cardiac death in Southern China. Medicine (Baltimore).

[13] Baumgartner H., De Backer J. (2020). The ESC clinical practice guidelines for the management of adult congenital heart disease 2020. Eur Heart J.

[14] Stout K.K., Daniels C.J., Aboulhosn J.A. (2019). 2018 AHA/ACC guideline for the management of adults with congenital heart disease: executive summary: a report of the American College of Cardiology/American Heart Association Task Force on Clinical Practice Guidelines. Circulation.

[15] Tso J., Turner C.G., Kim J.H. (2020). A hidden threat: anomalous aortic origins of the coronary arteries in athletes. Curr Treat Options Cardiovasc Med.

[16] Meijer F.M.M., Egorova A.D., Jongbloed M.R.M. (2021). The significance of symptoms before and after surgery for anomalous aortic origin of coronary arteries in adolescents and adults. Interact Cardiovasc Thorac Surg.

[17] Tonino P.A., De Bruyne B., Pijls N.H. (2009). Fractional flow reserve versus angiography for guiding percutaneous coronary intervention. N Engl J Med.

[18] Gotberg M., Christiansen E.H., Gudmundsdottir I.J. (2017). Instantaneous wave-free ratio versus fractional flow reserve to guide PCI. N Engl J Med.

[19] Mintz G.S., Nissen S.E., Anderson W.D. (2001). American College of Cardiology Clinical Expert Consensus Document on Standards for Acquisition, Measurement and Reporting of Intravascular Ultrasound Studies (IVUS). A report of the American College of Cardiology Task Force on Clinical Expert Consensus Documents. J Am Coll Cardiol.

[20] Grani C., Buechel R.R., Kaufmann P.A., Kwong R.Y. (2017). Multimodality imaging in individuals with anomalous coronary arteries. JACC Cardiovasc Imaging.

[21] Grani C., Kaufmann P.A., Windecker S., Buechel R.R. (2019). Diagnosis and management of anomalous coronary arteries with a malignant course. Interv Cardiol.

[22] Bigler M.R., Ueki Y., Otsuka T. (2020). Discrepancy between SPECT and dobutamine FFR in right anomalous coronary artery undergoing unroofing. Ann Thorac Surg.

[23] Driesen B.W., Warmerdam E.G., Sieswerda G.T. (2018). Anomalous coronary artery originating from the opposite sinus of Valsalva (ACAOS), fractional flow reserve- and intravascular ultrasound-guided management in adult patients. Catheter Cardiovasc Interv.

[24] Angelini P., Uribe C., Monge J. (2015). Origin of the right coronary artery from the opposite sinus of Valsalva in adults: characterization by intravascular ultrasonography at baseline and after stent angioplasty. Catheter Cardiovasc Interv.

[25] Gulati M., Levy P.D., Mukherjee D. (2021). 2021 AHA/ACC/ASE/CHEST/SAEM/SCCT/SCMR guideline for the evaluation and diagnosis of chest pain: a report of the American College of Cardiology/American Heart Association Joint Committee on Clinical Practice Guidelines. Circulation.

[26] Bigler M.R., Ashraf A., Seiler C. (2020). Hemodynamic relevance of anomalous coronary arteries originating from the opposite sinus of Valsalva—in search of the evidence. Front Cardiovasc Med.

[27] Bigler M.R., Kadner A., Raber L. (2022). Therapeutic management of anomalous coronary arteries originating from the opposite sinus of Valsalva: current evidence, proposed approach, and the unknowing. J Am Heart Assoc.

[28] McCray L.C., Fogwe D.T., Aggarwal K., Karuparthi P.R. (2019). Novel assessment of ischemia in patients with anomalous right coronary artery. JACC Case Rep.

[29] Abbara S., Blanke P., Maroules C.D. (2016). SCCT guidelines for the performance and acquisition of coronary computed tomographic angiography: a report of the Society of Cardiovascular Computed Tomography Guidelines Committee: Endorsed by the North American Society for Cardiovascular Imaging (NASCI). J Cardiovasc Comput Tomogr.

[30] Koppel C.J., Vliegen H.W., Bokenkamp R. (2022). The Leiden Convention coronary coding system: translation from the surgical to the universal view. Eur Heart J Cardiovasc Imaging.

[31] Shahoud J.S., Ambalavanan M., Tivakaran V.S. (2022).

[32] Cheezum M.K., Ghoshhajra B., Bittencourt M.S. (2017). Anomalous origin of the coronary artery arising from the opposite sinus: prevalence and outcomes in patients undergoing coronary CTA. Eur Heart J Cardiovasc Imaging.

[33] Cheezum M.K., Liberthson R.R., Shah N.R. (2017). Anomalous aortic origin of a coronary artery from the inappropriate sinus of Valsalva. J Am Coll Cardiol.

[34] Koppel C.J., Verheijen D.B.H., Kies P. (2022). A comprehensive analysis of the intramural segment in interarterial anomalous coronary arteries using computed tomography angiography. Eur Heart J Open.

[35] Lawton J.S., Tamis-Holland J.E., Bangalore S. (2022). 2021 ACC/AHA/SCAI guideline for coronary artery revascularization: a report of the American College of Cardiology/American Heart Association Joint Committee on Clinical Practice Guidelines. Circulation.

[36] Matsuo H., Kawase Y. (2016). FFR and iFR guided percutaneous coronary intervention. Cardiovasc Interv Ther.

[37] Knuuti J., Wijns W., Saraste A. (2020). 2019 ESC guidelines for the diagnosis and management of chronic coronary syndromes. Eur Heart J.

[38] Koppel C.J., Driesen B.W., de Winter R.J. (2021). The first multicentre study on coronary anomalies in the Netherlands: MuSCAT. Neth Heart J.

[39] Suryadinata R.V., Wirjatmadi B., Adriani M., Lorensia A. (2020). Effect of age and weight on physical activity. J Public Health Res.

[40] Takagi D., Nishida Y., Fujita D. (2015). Age-associated changes in the level of physical activity in elderly adults. J Phys Ther Sci.

[41] da Silva H.S., Nakamura F.Y., Papoti M., da Silva A.S., Dos-Santos J.W. (2021). Relationship between heart rate, oxygen consumption, and energy expenditure in futsal. Front Psychol.

[42] Van der Ploeg C.P., Dankelman J., Spaan J.A. (1995). Heart rate affects the dependency of myocardial oxygen consumption on flow in goats. Heart Vessels.

[43] Laurent D., Bolene-Williams C., Williams F.L., Katz L.N. (1956). Effects of heart rate on coronary flow and cardiac oxygen consumption. Am J Physiol.

[44] Christou D.D., Seals D.R. (2008). Decreased maximal heart rate with aging is related to reduced {beta}-adrenergic responsiveness but is largely explained by a reduction in intrinsic heart rate. J Appl Physiol (1985).

[45] Ozemek C., Whaley M.H., Finch W.H., Kaminsky L.A. (2017). Maximal heart rate declines linearly with age independent of cardiorespiratory fitness levels. Eur J Sport Sci.

[46] Laogun A.A., Gosling R.G. (1982). In vivo arterial compliance in man. Clin Phys Physiol Meas.

[47] Blomjous M.S.H., Budde R.P.J., Bekker M.W.A. (2021). Clinical outcome of anomalous coronary artery with interarterial course in adults: single-center experience combined with a systematic review. Int J Cardiol.

[48] Almeida I., Santos H., Miranda H. (2020). Cardiac arrest due to an anomalous aortic origin of a coronary artery: are older patients really safe?. Rev Bras Ter Intensiva.

[49] Jennings B.R., van Gaal W.J., Banning A.P. (2007). Extrinsic compression of an anomalous right coronary artery causing cardiac arrest. Heart.

[50] Bruls S., Durieux R., Gach O., Lancellotti P., Defraigne J.O. (2020). Sudden cardiac death revealed by an anomalous origin of the right coronary artery from the left sinus of Valsalva. Ann Thorac Surg.

[51] Jaggers J., Lodge A.J. (2005). Surgical therapy for anomalous aortic origin of the coronary arteries. Semin Thorac Cardiovasc Surg Pediatr Card Surg Annu.

[52] Padalino M.A., Franchetti N., Hazekamp M. (2019). Surgery for anomalous aortic origin of coronary arteries: a multicentre study from the European Congenital Heart Surgeons Association. Eur J Cardiothorac Surg.

[53] Gharibeh L., Rahmouni K., Hong S.J., Crean A.M., Grau J.B. (2021). Surgical techniques for the treatment of anomalous origin of right coronary artery from the left sinus: a comparative review. J Am Heart Assoc.

[54] Lee S.E., Yu C.W., Park K. (2016). Physiological and clinical relevance of anomalous right coronary artery originating from left sinus of Valsalva in adults. Heart.

