# Supplementary material for: Anomalous Aortic Origin of the Right Coronary Artery: Invasive Haemodynamic Assessment in Adult Patients With High-Risk Anatomic Features
Source: CJC Pediatr Congenit Heart Dis. 2023 Mar 8;2(3):124–33. doi: 10.1016/j.cjcpc.2023.03.001 (PMC10642095; doi:10.1016/j.cjcpc.2023.03.001)
Supplement: Supplemental Table [file mmc1.pdf]

Supplemental Table S1. IVUS measurements at baseline and after adrenaline infusion.

	At baseline				Adrenaline infusion				
**Study #**	**Height (mm)**	**Width (mm)**	**Surface area (mm^2)**	**W/H ratio**	**Height (mm)**	**Width (mm)**	**Surface area (mm^2)**	**W/H ratio**	**W/H ratio adrenaline / W/H ratio baseline**
**1**	5.5	1.9	9.0	0.35	5.7	1.9	8.3	0.33	1.0
**2**	5.1	1.9	8.0	0.37	5.4	2.4	10.3	0.44	1.2
**3**	3.9	2.6	8.6	0.67	4.1	2.4	8.3	0.59	0.9
**4**	3.4	1.7	4.7	0.50	4.0	1.5	4.5	0.38	0.8
**5**	4.8	2.5	9.8	0.52	4.8	2.5	9.8	0.52	1.0
**6**	4.8	1.9	8.3	0.40	6.3	2.4	10.9	0.38	1.0
**7**	3.6	1.8	4.5	0.50	3.9	1.6	4.4	0.41	0.8
**8**	4.5	2.1	7.0	0.47	4.4	2.0	6.6	0.45	1.0

Abbreviations: NTG, nitroglycerine; W/H ratio, width/height ratio.
